# Examining contemporaneous and temporal associations of real-time suicidal ideation using network analysis

**DOI:** 10.1017/S003329172400151X

**Published:** 2024-09

**Authors:** Liia M. M. Kivelä, Eiko I. Fried, Willem van der Does, Niki Antypa

**Affiliations:** 1 Department of Clinical Psychology, Institute of Psychology, Leiden University, Leiden, The Netherlands; 2 Leiden University Treatment and Expertise Center (LUBEC), Leiden, The Netherlands

**Keywords:** acquired capability, ecological momentary assessment, hopelessness, shame, suicide

## Abstract

**Background:**

Suicidal ideation arises from a complex interplay of multiple interacting risk factors over time. Recently, ecological momentary assessment (EMA) has increased our understanding of factors associated with real-time suicidal ideation, as well as those predicting ideation at the level of hours and days. Here we used statistical network methods to investigate which cognitive-affective risk and protective factors are associated with the temporal dynamics of suicidal ideation.

**Methods:**

The SAFE study is a longitudinal cohort study of 82 participants with current suicidal ideation who completed 4×/day EMA over 21 days. We modeled contemporaneous (*t*) and temporal (*t +* 1) associations of three suicidal ideation components (passive ideation, active ideation, and acquired capability) and their predictors (positive and negative affect, anxiety, hopelessness, loneliness, burdensomeness, and optimism) using multilevel vector auto-regression models.

**Results:**

Contemporaneously, passive suicidal ideation was positively associated with sadness, hopelessness, loneliness, and burdensomeness, and negatively with happiness, calmness, and optimism; active suicidal ideation was positively associated with passive suicidal ideation, sadness, and shame; and acquired capability only with passive and active suicidal ideation. Acquired capability and hopelessness positively predicted passive ideation at *t +* 1, which in turn predicted active ideation; acquired capability was positively predicted at *t +* 1 by shame, and negatively by burdensomeness.

**Conclusions:**

Our findings show that systematic real-time associations exist between suicidal ideation and its predictors, and that different factors may uniquely influence distinct components of ideation. These factors may represent important targets for safety planning and risk detection.

## Introduction

Suicidal ideation is influenced by multiple interacting risk and protective factors over time (de Beurs et al., 2021; Franklin et al., 2017; Goldston et al., 2016). Some risk factors, such as sociodemographic characteristics and childhood adversity, may exert their influences over one's lifetime (Nock et al., 2008), but are not useful in assessing imminent risk. The influence of other risk factors, such as stressful life events, although more temporally limited (Howarth et al., 2020), has shown poor sensitivity in identifying those most at risk. Some other factors, such as abrupt changes in sleep or affect (Allen, Nelson, Brent, & Auerbach, 2019), may have even more temporally specific effects, and help in identifying those with heightened imminent risk. Collectively, these latter factors are known as *acute warning signs* of suicide (Rudd et al., 2006), i.e. factors that are associated with suicide risk in the short-term. The aim of the present study was to model real-time data on suicidal ideation and its warning signs to uncover patterns that characterize short-term changes in suicidal ideation.

Although familiar to healthcare professionals, acute warning signs have been given relatively little research attention (Rudd, 2008), probably because they are sometimes fleeting and therefore quite difficult to measure. However, the development of *ecological momentary assessment* (*EMA*) (Davidson, Anestis, & Gutierrez, 2017; Shiffman, Stone, & Hufford, 2008) and its increased application in suicide research have facilitated a stronger focus on these warning signs. EMA, which refers to real-time data collection methods in individuals’ natural environments, allows for a fine-grained examination of the temporal effects of suicidal ideation, as well as its risk and protective factors (de Beurs, Kirtley, Kerkhof, Portzky, & O'Connor, 2015; Kivelä, van der Does, Riese, & Antypa, 2022; Nock, 2016). EMA data may be used to examine momentary correlates of high or low suicidal ideation or to build prediction models that aim to forecast changes in suicidal ideation in the subsequent hours and days. Increased attention on this acute time frame is crucial, as it has previously gone largely neglected (de Beurs et al., 2015; Franklin et al., 2017; Glenn & Nock, 2014). Now, a more detailed examination of the temporal dynamics of suicidal ideation is needed, with a shift to identifying *state* rather than trait predictors of suicidal ideation.

EMA research on suicidal ideation allows researchers to focus on this clinically relevant timeframe (hours, days), and has already provided some new insights. We recently reviewed 23 studies that used EMA to assess suicidal ideation (Kivelä et al., 2022). These studies have demonstrated that many known long-term suicide risk factors are also momentary correlates of suicidal ideation. Among these are contextual factors (such as being alone) (Husky et al., 2017; Nock, Pristein, & Sterba, 2009), interpersonal conflict (Kaurin, Dombrovski, Hallquist, & Wright, 2020; Nock et al., 2009), maladaptive coping and rumination (Hallard, Wells, Aadahl, Emsley, & Pratt, 2021), increased negative affect (Armey, Brick, Schatten, Nugent, & Miller, 2020; Husky et al., 2017), as well as hopelessness, burdensomeness and loneliness/thwarted belongingness (Czyz, Horwitz, Arango, & King, 2019; Hallensleben et al., 2019; Kleiman et al., 2017). Fewer studies have examined prospective (short-term) associations with suicidal ideation. Consequently, few temporal predictors of ideation have been established. Suicidal ideation itself appears to be strongly autocorrelated within-day (Kleiman et al., 2017), but evidence for other temporal predictors is scarce, inconsistent, and requires further work. For example, hopelessness and burdensomeness (Hallensleben et al., 2019), negative affect (Armey et al., 2020; Victor, Scott, Stepp, & Goldstein, 2019), active coping (Stanley et al., 2021), as well as sleep duration (Littlewood et al., 2019) may be predictive of suicidal ideation in the short-term.

Further, only a limited number of EMA studies have clearly distinguished between different components of suicidal ideation. These include passive and active suicidal ideation (Wastler et al., 2023), as well as *acquired capability*, referring to increased internal preparedness for suicidal behavior, encompassing decreased fearlessness about death and increased pain tolerance (Van Orden et al., 2010). The identification of predictors of active suicidal ideation and acquired capability may be especially important, as these constructs are more closely related to the transition from ideation to action (Díaz-Oliván, Porras-Segovia, Barrigón, Jiménez-Muñoz, & Baca-García, 2021; Van Orden et al., 2010). From the few studies that have aimed to disentangle these components, differential findings have emerged. Perceived burdensomeness was found to concurrently associated with passive, but not active, suicidal ideation, while hopelessness, depressed mood, and thwarted belongingness were related to both active and passive ideation (Hallensleben et al., 2019). Finally, higher daily levels of active ideation predicted higher acquired capability ratings at the end of the day (Spangenberg, Glaesmer, Hallensleben, Rath, & Forkmann, 2019). These findings illustrate the importance of separating different components of suicidal ideation.

An emerging modeling technique, namely *network analysis*, allows for the synthesis of this information in a manner that enables researchers to model the complexity of systems with multiple outcomes and multiple interacting risk and protective factors over time (Borsboom et al., 2021; Bringmann et al., 2013; de Beurs, 2017; Fried & Cramer, 2017). As such, network modeling can address both of the current challenges in EMA suicide research: account for the complexity in both predictors and outcomes, and help explore short-term, temporal associations. Network models in time-series data can estimate potentially bidirectional associations not only between suicidal ideation and its risk factors but between different suicidal ideation outcomes as well, in order to observe the full extent of both direct and indirect influences on suicidal ideation. Further, in network models, risk factors such as loneliness or hopelessness reflect pieces in the greater network of the symptomatology of suicidal ideation rather than simply being potential *causes* of suicidal ideation. In other words, suicidal ideation can both be influenced by, and further influence, these risk factors, and network modeling may be used to visualize these complex, bidirectional temporal relationships.

Network analysis is most often applied to complex time-series data, such as those collected via EMA. So far, only one study has applied network analysis to such data on suicidal ideation. Among 74 psychiatric inpatients who completed six days of EMA with 10 prompts per day, contemporaneous (i.e. concurrent) associations were found between suicidal ideation and hopelessness, thwarted belongingness, burdensomeness, positive and negative affect, and anxiety; however, only burdensomeness emerged as a significant temporal predictor of within-day suicidal ideation (Rath et al., 2019). As such, current EMA studies of real-time suicidal ideation (and network models emerging from such data) have not yet established robust short-term temporal predictors of ideation. Further, the distinction between different components of suicidal ideation, and how different risk and protective factors may differentially associate with these outcomes, have not been considered in such models.

The aim of the present study was to further investigate the temporal dynamics of different components of suicidal ideation. We applied network analysis to EMA data to examine how cognitive-affective risk and protective factors (incl. positive and negative affect, anxiety, hopelessness, loneliness, burdensomeness, and optimism) are interconnected, and how they interact in the prediction of suicidal ideation (passive ideation, active ideation, and acquired capability) in the short-term. While the potential range of risk and protective factors impacting suicidal ideation is vast (de Beurs et al., 2021; Franklin et al., 2017; Goldston et al., 2016), past EMA studies have demonstrated that maladaptive cognitions (hopelessness, loneliness, burdensomeness) and affect variables specifically appear to form the most robust associations with real-time suicidal ideation (Kivelä et al., 2022). According to the Interpersonal Psychological Theory of Suicide (IPTS) (Van Orden et al., 2010), hopelessness, loneliness and burdensomeness are crucial for the development of suicidal ideation, and are also interconnected with other established risk factors (Kleiman, Law, & Anestis, 2014). For example, a negative cognitive style (e.g. hopelessness-proneness), may be associated both with specific negative attributions (‘I am alone’, ‘I am a burden’), as well as other negative affective sequale (feelings of shame, anger, sadness, etc.). Cognition and affect interact; affect can influence cognition and similarly, cognitions may trigger affective responses (Duncan & Barrett, 2007), resulting in bidirectional associations with suicidal ideation. Considering that no previous study has examined the combined real-time associations between these variables in relation to different components of suicidal ideation, we adopted an explorative framework and did not specify *a priori* predictions of differential associations with passive and active suicidal ideation, and acquired capability.

## Methods

### Design

Data were collected in the SAFE study, a longitudinal cohort study in individuals with current suicidal ideation, who completed 21 days of EMA 4×/day.

### Ethical approval

The study was approved by the Medical Ethics Committee – Leiden, Den Haag, Delft (The Netherlands) (METC-LDD) on 24.04.2020 (NL71510.058.19).

### Participants

Participants (*N* = 82) for the study were adults with a history of a suicide attempt and/or active suicidal ideation in the past year (Columbia Suicide Severity Rating Scale (CSSRS) (Posner et al., 2011) score of > = 3, or > = 2 if ideation was present in the past two months). All participants endorsed past 12-month active suicidal ideation on the CSSRS, of which 26 (32%) reported that this ideation was still present within the past two months. All participants who endorsed a past 12-month suicide attempt (*n* = 17, 21%) also reported past 12-month active ideation. Additional inclusion criteria comprised proficiency in written and spoken English and/or Dutch, being registered with a Dutch general practitioner (GP), and possession of an iOS or Android compatible smartphone. Exclusion criteria were a (current) diagnosis of bipolar disorder, a psychotic disorder, or severe substance dependence, or any other intellectual or physical impairment that would have prevented the participant from adequately following the study procedures. More information on study proceedings may be found in Kivelä, Fiß, van der Does, and Antypa (2023).

### Measures and procedure

Participants were recruited through fliers distributed in the community (incl. social media), as well as collaborating mental health care providers in the area. Fliers included a QR code directing participants to the study website, where they could access full study information, and fill in a ‘self-test’ to check their eligibility for the study. Interested participants could then fill in a contact form to be invited for an intake interview either on location (Leiden) or online. A total of 209 participants signed up for the study and were invited for the intake interview, of which 90 attended the interview.

During the intake, participants received information about the study and their role as a participant, and after signing written informed consent, completed a semi-structured interview covering information on their sociodemographic characteristics, and medical and psychiatric history. The MINI Neuropsychiatric Interview (v. 5) (Sheehan et al., 1998) and the Structured Clinical Interview for DSM-5 Personality Disorders subscale for Borderline Personality Disorder (SCID-PD-BPD) were used to establish current diagnoses, and an adapted version of the CSSRS (Posner et al., 2011) was used to assess the participants' past-year history of suicidal ideation, as well as their lifetime history of suicide attempts. Following the interview, eight participants were excluded (*n* = 2 because they declined to participate, and *n* = 6 on the basis of inclusion/exclusion criteria, see Kivelä et al., 2023 for more information on participant flow). Following eligibility assessment, and prior to receiving instructions for the EMA, a personalized suicide safety plan was drafted for each participant detailing their resources in the case of a suicidal crisis. Finally, participants were instructed on how to download the EMA app (created by Ethicadata.com), and the use of the app was illustrated by means of a demo questionnaire and written instructions provided to the participants.

During 21 days, participants received four daily (scheduled) EMA prompts on a signal-contingent, pseudo-random schedule. Prompts were sent out at randomized times within the windows of 7am–9am, 12pm–2pm, 4pm–6pm, and 8pm–10pm. Following the alert, participants had 180 min to fill in the morning assessment, and 120 min to fill in the afternoon and evening assessments. Reminder alerts were sent out 30 min after the initial alert in case the EMA had not yet been completed. Additionally, participants could self-initiate (additional) entries at any time during the EMA period. The EMA items are presented in Table 1. Passive suicidal ideation was defined as the mean of two items, active suicidal ideation as the mean of two items, and acquired capability as the mean of three items.

**Table 1.** Ecological momentary assessment (EMA) items

Parameter	Item	Scale
Suicidal ideation		
Passive ideation	At the moment, how strong is your desire to live?	0 (none) – 10 (very strong)*
	At the moment, how strong is your desire to die, or go to sleep and not wake up?	0 (none) – 10 (very strong)
Active ideation	At the moment, do you actually have thoughts of killing yourself?	0 (not at all) – 10 (very much)
	At the moment, how strong is your intention to act on these thoughts?	0 (none) – 10 (very strong)
Acquired capability	At the moment, how much can you resist the urge to kill yourself?	0 (not at all) – 10 (very much)*
	At the moment, how afraid are you of dying?	0 (not at all) – 10 (very much)*
	At the moment, how afraid are you of the pain associated with dying?	0 (not at all) – 10 (very much)*
Happy	At the moment, how happy do you feel?	0 (not at all) – 10 (very much)
Calm	At the moment, how calm do you feel?	0 (not at all) – 10 (very much)
Sad	At the moment, how sad do you feel?	0 (not at all) – 10 (very much)
Anxious	At the moment, how anxious do you feel?	0 (not at all) – 10 (very much)
Angry	At the moment, how angry do you feel?	0 (not at all) – 10 (very much)
Guilty	At the moment, how guilty do you feel?	0 (not at all) – 10 (very much)
Ashamed	At the moment, how ashamed do you feel?	0 (not at all) – 10 (very much)
Hopeless	At the moment, how hopeless do you feel?	0 (not at all) – 10 (very much)
Optimistic	At the moment, how optimistic do you feel?	0 (not at all) – 10 (very much)
Lonely	At the moment, how lonely do you feel?	0 (not at all) – 10 (very much)
Burdensome	At the moment, I feel like I'm a burden to others in my life.	0 (not at all) – 10 (very much)

*Positively worded items were reverse coded so that higher scores on all items reflect more severe suicidal ideation.

### Statistical analysis

All analyses were conducted in R (version 4.0.2) using the *mlVAR* package (Epskamp, Deserno, & Bringmann, 2021) for fitting multilevel vector autoregression models. The R session and package information can be found in the Supplementary material. Assumptions for *mlVAR* models include equidistant observations, stationarity, and multivariate normality (Bringmann et al., 2013). To establish equidistant observations, we only estimated associations between successive observations within the same day (i.e. excluding associations between the last observation of day *d* and the first observation of the subsequent day *d* *+* *1*, which would include a longer time lag than the other observations which were approximately equally spaced within the day). We examined stationarity, i.e. the assumption that the means of all variables for all participants remain stable over time, using the Kwiatkowski–Phillips–Schmidt–Shin unit root test (KPSS) (Kwiatkowski, Phillips, Schmidt, & Shin, 1992); the test indicated that the assumption was met for most variables, for most participants. Happy was detrended for 25% of the participants, Calm for 23%, Sad for 20%, Anxious for 22%, Angry for 18%, Guilty for 32%, Shame for 32%, Hopeless for 24%, Optimistic for 24%, Loneliness for 15%, Burdensomeness for 32%, Passive suicidal ideation for 30%, Active suicidal ideation for 27%, and Acquired capability for 25% participants. Proportion of detrended time-series is similar to that in other EMA studies (see e.g., Jongeneel et al., 2020). Detrending was applied to transform each variable time series for each participant in which the assumption was violated, whereby the non-stationary time series was replaced with the participant's within-person mean (as previously done by e.g. Jongeneel et al., 2020). The Kolmogorov–Smirnov test was used to assess multivariate normality; all variables violated (*p* < 0.001) the assumption, as is often the case in EMA data (see e.g. Veenman et al., 2024). While violations of normality do not prevent the fitting of VAR models, they may reduce the power to detect small relations.

Prior to fitting the models, we examined potential multicollinearity between passive ideation, active suicidal ideation, and acquired capability. All variance inflation factor (VIF) values were < = 3 and tolerance = >0.30, indicating no multicollinearity. We used the *mlVAR* package to estimate (1) a contemporaneous model that presents concurrent associations between all variables at time *t*, and (2) a temporal model with a time lag to estimate associations between two subsequent assessments (*t* and *t* *+* *1*). In the contemporaneous model, all associations are controlled for the contemporaneous effects of all other variables in the model, as well as temporal effects and autocorrelations of all variables. In the temporal model, all associations are controlled for the temporal effects of the other variables in the model (i.e. unique partial contributions of each variable are estimated). We used orthogonal estimation, which is better suited for models with a larger number of variables (Epskamp et al., 2021). The *lmer* estimation method (which uses sequential univariate multilevel estimation) was used for all models. Results were visualized using the *qgraph* package (Epskamp, Cramer, Waldorp, Schmittmann, & Borsboom, 2012); the network graphs present associations (edges) between variables (nodes) whereby the thickness of the edges indicates the strength of the association, and the color of the edges the direction of the association (*dashed red:* negative association; *blue:* positive association). Significance for all analyses was determined at alpha = 0.05.

## Results

### Data exploration

The full EMA dataset consisted of 5400 observations, nested within 82 participants, and 21 days. Participants completed 66 surveys on average (median = 70, range = 16–88). After excluding participants with less than 20 observations, in line with guidelines for fitting *mlVAR* models (Epskamp et al., 2021), 5349 observations nested within 79 participants, and 21 days remained. Participants on average filled in 65 of the 81 scheduled alerts (range 22–81, total *k* = 5145), as well as three additional entries (range 0–13, total *k* = 204), resulting in a total of 68 entries per participant on average (range 24–88, total *K* = 5349). For fitting the contemporaneous (*t*) network model, we used all 5349 (*N* = 79) individual observations. For the temporal (*t* *+* *1*) network model, we included 3415 (*N* = 79) pairs of adjacent within-day observations (i.e. excluding any pairs of observations broken up by either missing data or transitions between days). Table 2 presents intra-individual means and standard deviations for all study variables as measured with the EMA. All participants indicated at least one observation of passive suicidal ideation (mean % of non-zero ratings = 91, range 3–100). Seventy-two participants (91%) additionally indicated at least one observation of both active suicidal ideation and acquired capability during the study period (mean % of non-zero ratings = 41 range 1–100).

**Table 2.** Intra-individual means and standard deviations

	Intra-individual	
	M	s.d.
Passive suicidal ideation	3.74	1.36
Active suicidal ideation	1.22	0.94
Acquired capability	2.10	1.46
Happy	4.90	1.58
Calm	5.30	1.81
Sad	3.54	1.98
Anxious	3.60	1.95
Angry	1.83	1.77
Guilty	2.80	1.67
Ashamed	2.71	1.61
Hopeless	3.76	1.87
Optimistic	4.18	1.59
Lonely	3.67	1.90
Burdensome	3.77	1.50

*Note:* Range for all variables 0–10.

### Sample characteristics

The sample (*N* = 79) was primarily female (80%), with the remaining participants identifying either as male (11%) or non-binary/trans (9%). The mean age of the sample was 27 (s.d. = 8.6). The sample comprised Dutch (54%) and other nationals (46%). The most prevalent current (past month) diagnoses were major depressive disorder (51%) and other depressive disorders (28%), anxiety disorders (56%), post-traumatic stress disorder (23%), autism spectrum disorder (18%), and borderline personality disorder (15%). Current psychiatric medication use was reported by 60% of the sample, and 43% had a history of at least one prior suicide attempt. More detailed information on sample characteristics may be found in Kivelä et al. (2023).

### Contemporaneous associations with passive and active suicidal ideation and acquired capability

**Figure 1.**
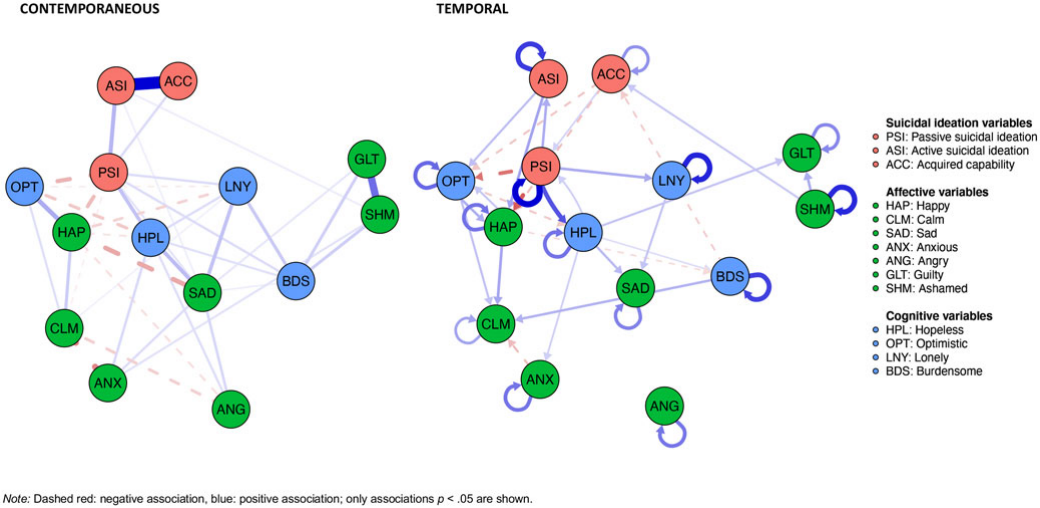
Contemporaneous (*t*) (left) and temporal (*t* + 1) (right) associations with passive and active suicidal ideation and acquired capability.

In the contemporaneous model (Fig. 1, left), passive suicidal ideation was positively associated with sadness (*r* = 0.07, *p* < 0.001), hopelessness (*r* = 0.16, *p* < 0.001), loneliness (*r* = 0.11, *p* < 0.001), and burdensomeness (*r* = 0.09, *p* < 0.001), and negatively associated with happiness (*r* = –0.18, *p* < 0.001), calmness (*r* = –0.05, *p* = 0.017), and optimism (*r* = –0.20, *p* < 0.001). Active suicidal ideation was positively associated with passive suicidal ideation (*r* = 0.20, *p* < 0.001), sadness (*r* = 0.05, *p* = 0.004) and shame (*r* = 0.05, *p* = 0.028). Acquired capability was positively associated with passive suicidal ideation (*r* = 0.10, *p* < 0.001) and active suicidal ideation (*r* = 0.69, *p* < 0.001).

### Temporal associations with passive and active suicidal ideation and acquired capability

In the temporal model (Fig. 1, right), passive suicidal ideation (*r* = 0.30, *p* < 0.001), active suicidal ideation (*r* = 0.23, *p* < 0.001), and acquired capability (*r* = 0.13, *p* = 0.001) all exhibited significant positive autocorrelations. Increased hopelessness (*r* = 0.06, *p* = 0.003) and acquired capability (*r* = 0.13, *p* = 0.001) were predictive of higher levels of passive ideation at the subsequent time point. Passive ideation in turn predicted increased active ideation (*r* = 0.09, *p* = 0.002), hopelessness (*r* = 0.20, *p* < 0.001) and loneliness (*r* = 0.10, *p* = 0.006), and decreased happiness (*r* = –0.19, *p* < 0.001) and optimism (*r* = –0.16, *p* < 0.001) at the subsequent assessment point. None of the other variables (except for passive ideation, see above) prospectively predicted active ideation at the subsequent time point. However, active ideation in turn predicted increased happiness (*r* = 0.11, *p* = 0.003) and optimism (*r* = 0.07, *p* = 0.033) at the subsequent assessment point. Increased shame (*r* = 0.06, *p* = 0.004) and decreased burdensomeness (*r* = –0.06, *p* = 0.021) were associated with heightened acquired capability at the subsequent time point. Acquired capability in turn predicted decreased happiness (*r* = –0.07, *p* = 0.033) and optimism (*r* = –0.07, *p* = 0.017) at the subsequent assessment point.

## Discussion

### Passive suicidal ideation

Momentary passive suicidal ideation correlated with sadness, hopelessness, loneliness, and burdensomeness, in line with prior literature (Armey et al., 2020; Czyz et al., 2019; Hallensleben et al., 2019; Husky et al., 2017; Kleiman et al., 2017). It has previously been shown that perceived burdensomeness associated only with passive and not active suicidal ideation (Hallensleben et al., 2019). Here, we found all three constructs of the Interpersonal Theory of Suicide (IPTS; Van Orden et al., 2010) (i.e. hopelessness, loneliness, and burdensomeness) to associate only with passive, but not active, ideation. Passive suicidal ideation was also associated with reduced happiness, calmness, and optimism, in line with prior reports of decreased positive affect (and happiness specifically) relating to momentary suicidal ideation (Husky et al., 2017; Rath et al., 2019). Our findings add to this literature by demonstrating concurrent, negative associations with another facet of positive affect: calmness. Indeed, retrospective reports by clinicians and family members have long described that individuals often appear agitated in the days preceding suicide (Sani et al., 2011). In line with our findings on momentary optimism, another study previously found positive thinking-based coping to decrease suicidal ideation in daily life (Stanley et al., 2021).

Passive ideation was prospectively predicted by increased hopelessness and acquired capability. Using EMA data, only one previous study has highlighted hopelessness as a prospective predictor of ideation: among psychiatric inpatients, hopelessness predicted both passive and active ideation within-day (Hallensleben et al., 2019). Meanwhile, others did not establish hopelessness as a prospective (short-term) predictor of ideation (Czyz et al., 2019; Kleiman et al., 2017). However, of note is that both studies examined active ideation only. Our findings therefore add to this literature by demonstrating that hopelessness may be uniquely associated with passive suicidal ideation. We propose that the different operationalization of suicidal ideation in prior studies may partly explain the contradictory findings.

Acquired capability also prospectively predicted increased passive ideation, which in turn predicted active ideation. It may be expected that a passive lack of will to live or a wish to die will over time develop into more concrete thoughts about death and/or suicide (Van Orden et al., 2010). Our findings illustrate that this transition may occur relatively quickly (in approx. 4 h), although it is important to note that our sample was composed of individuals with a long-term (months, years) history of suicidal ideation. Hence, it is unlikely that someone experiencing first-time passive ideation would progress to active ideation so rapidly, but rather our data reflects moment-to-moment changes in individuals who are already familiar with suicidal states.

### Active suicidal ideation

Active ideation was concurrently associated only with sadness and shame (excluding the triadic associations between passive and active ideation, and acquired capability). Shame is specifically associated with the lethality of suicide attempts (Van Orden et al., 2010), which may explain its unique association with active suicide ideation. Further, shame is closely related to other forms of (non-suicidal) self-harm (Sheehy et al., 2019). This is proposed to result from the strong overlap between shame, self-hatred and the need to punish oneself (Sheehy et al., 2019) – or perhaps in the case of suicidal behavior, to completely eliminate oneself.

Somewhat paradoxically, active ideation also prospectively predicted *increased* happiness and optimism. This is in contrast to our findings on passive ideation and acquired capability, which were followed by negative mood consequences. We speculate that this pattern simply reflects the passing of a suicidal crisis leading to feelings of relief. However, others reporting similar findings propose that some people engage in suicidal thinking as a way of regulating their affect, and hence experience suicidal thoughts as comforting (Kleiman et al., 2018). While a subset of patients does report comfort from ideation (Crane et al., 2014), most people describe their suicidal thoughts as distressing, as also demonstrated by a previous EMA study which found increased negative affect following instances of suicidal thinking (Al-Dajani & Uliaszek, 2021). However, in case suicidal thinking does serve this relief function, it appears that it is active, rather than passive suicidal ideation that produces this effect. This finding also fits within the framework of suicide representing an escape from psychological pain (Baumeister, 1990).

### Acquired capability

Acquired capability was concurrently associated only with passive and active suicidal ideation. The finding that acquired capability was more strongly associated with active rather than passive ideation supports the notion that acquired capability and active ideation are more closely related, and together may be more influential in predicting suicidal acts (Van Orden et al., 2010). The lack of other concurrent associations is also in line with the IPTS, which posits that risk factors such as hopelessness and loneliness are crucial for the development of suicidal ideation, but are not necessarily directly related to acquired capability (Van Orden et al., 2010).

We did, however, find that increased shame and decreased burdensomeness prospectively predicted acquired capability. Shame and burdensomeness have many related characteristics. While shame is considered the emotion perhaps most related to self-hatred (Sheehy et al., 2019), the concept of burdensomeness includes beliefs such as that ‘the self is so flawed as to be a liability on others’ (Van Orden et al., 2010, p. 12). Meanwhile, burdensomeness is more related to the perception of self in relation to others, while shame is more self-directed. Therefore, through repeated negative experiences with others, feelings of burdensomeness may over time become internalized into deeper feelings of shame and self-hatred. This may explain why further down in the pathway to suicide the role of burdensomeness may be reduced, while shame takes a more central role (Van Orden et al., 2010).

### Limitations

Certain limitations should be considered when interpreting these findings. First, established power calculations for multilevel VAR models are lacking and it remains to be determined how many participants and time points are needed to obtain precise estimates We acknowledge power as a potential limitation and urge future research to replicate these findings in larger samples. Second, due to the nature of network models that are highly parameterized, we did not include additional predictors in our models to balance comprehensiveness with statistical power. In line with the systems approach to understanding psychopathology, suicidal ideation is a multifaceted phenomenon, for which any one risk factor is likely to have only limited explanatory or predictive power (Fried, 2022; Fried & Robinaugh, 2020). We hope that future research identifies ways to obtain comprehensive system estimates for suicidal ideation, considering cognitive-affective (e.g. sadness), contextual (e.g. social contact), and behavioral (e.g. coping) components. Third, we must work towards a better understanding of the timeline within which different factors affect suicidal ideation in order to inform study designs: how do we space EMA prompts to optimally predict suicidal ideation? In our analyses, we observed relatively more concurrent rather than temporal associations. However, just because some variables did not emerge as temporal predictors does not necessarily mean that they are not prospectively associated with suicidal ideation – it only means that they are not associated with ideation within the very specific time frame (approx. 4 h) that we had between observations in our study. Instead, it is possible that some factors, such as sadness, may exert their influences much more rapidly, in which case these associations would emerge in the contemporaneous models. Other factors, such as shame, may need longer to result in suicidal ideation, and accumulate over time before their effects become apparent. Finally, our sample was predominantly female and skewed younger in age distribution. The influence of many of the examined risk factors may differ as a function of sociodemographic characteristics (e.g. factors such as loneliness may differentially affect different age groups and genders, see e.g. Boehlen et al., 2022). Gender differences also exist in interpersonal sensitivity, such as the experience of shame (Nyström, Kjellberg, Heimdahl, & Jonsson, 2018).

### Clinical implications

Our findings on the differential associations between suicide risk factors on one hand, and passive and active suicidal ideation as well as acquired capability on the other, have clinical relevance. First, we observed unique associations of hopelessness, loneliness, and burdensomeness with passive suicidal ideation, indicating that negative cognitive attributional styles may be more central for the foundational development of passive suicidal ideation. These factors may therefore represent important targets in the long-term therapeutic management of suicidality (Van Orden, Talbot, & King, 2012). However, our finding indicating that shame specifically was uniquely associated with active suicidal ideation, and further predicted short-term increases in acquired capability, indicates that for acute risk management, targeting other affective processes may be more crucial. Shame encompasses intense feelings of embarrassment and self-hatred (Lester, 1997), and may therefore represent an especially aversive internal state that is more likely to lead to active thoughts and preparedness to ‘escape’ the shame-inducing experience (Baumeister, 1990). Our findings indicate that shame-reduction techniques (Goffnett, Liechty, & Kidder, 2020) may also benefit the treatment of patients with suicidal ideation.

## Conclusions

We observed differential associations of risk factors with passive and active suicidal ideation and acquired capability. Hopelessness, loneliness, and burdensomeness were uniquely associated with passive but not active suicidal ideation, and shame with active suicidal ideation and acquired capability. Overall, our findings illustrate how ecological momentary assessment and network analysis may be used to better understand and visualize the cognitive-affective landscape from which suicidal ideation may emerge in real time. Future research using real-time assessments should aim to further distinguish the various risk and protective factors that may differentially characterize passive, active ideation, and acquired capability outcomes. A clinical implication of our findings is that targeting shame may be especially relevant for suicide prevention, considering its unique contribution to explaining not only short-term increases in active suicidal ideation but also the preparedness for suicidal acts.

## Supporting information

Kivelä et al. supplementary material Kivelä et al. supplementary material

## Supplementary material

The supplementary material for this article can be found at https://doi.org/10.1017/S003329172400151X.

## References

[ref1] Al-Dajani, N., & Uliaszek, A. A. (2021). The after-effects of momentary suicidal ideation: A preliminary examination of emotion intensity changes following suicidal thoughts. Psychiatry Research, 302, 114027. 10.1016/j.psychres.2021.114027 34139594

[ref2] Allen, N. B., Nelson, B. W., Brent, D., & Auerbach, R. P. (2019). Short-term prediction of suicidal thoughts and behaviors in adolescents: Can recent developments in technology and computational science provide a breakthrough? Journal of Affective Disorders, 250, 163–169. 30856493 10.1016/j.jad.2019.03.044 PMC6481940

[ref3] Armey, M. F., Brick, L., Schatten, H. T., Nugent, N. R., & Miller, I. W. (2020). Ecologically assessed affect and suicidal ideation following psychiatric inpatient hospitalization. General Hospital Psychiatry, 63, 89–96. 30297091 10.1016/j.genhosppsych.2018.09.008 PMC6581626

[ref4] Baumeister, R. F. (1990). Suicide as escape from self. Psychological Review, 97(1), 90–113. 10.1037/0033-295X.97.1.90 2408091

[ref5] Boehlen, F. H., Maatouk, I., Friederich, H.-C., Schoettker, B., Brenner, H., & Wild, B. (2022). Loneliness as a gender-specific predictor of physical and mental health-related quality of life in older adults. Quality of Life Research, 31(7), 2023–2033. 10.1007/s11136-021-03055-1 34859354 PMC9188519

[ref6] Borsboom, D., Deserno, M. K., Rhemtulla, M., Epskamp, S., Fried, E. I., McNally, R. J., … Waldorp, L. J. (2021). Network analysis of multivariate data in psychological science. Nature Reviews Methods Primers, 1(1), 58. 10.1038/s43586-021-00055-w

[ref7] Bringmann, L. F., Vissers, N., Wichers, M., Geschwind, N., Kuppens, P., Peeters, F., … Tuerlinckx, F. (2013). A network approach to psychopathology: New insights into clinical longitudinal data. PLoS ONE, 9(4), e96588. 10.1371/journal.pone.0060188 PMC3617177 23593171

[ref8] Crane, C., Barnhofer, T., Duggan, D. S., Eames, C., Hepburn, S., Shah, D., & Williams, J. M. G. (2014). Comfort from suicidal cognition in recurrently depressed patients. Journal of Affective Disorders, 155, 241–246. 10.1016/j.jad.2013.11.006 24289891 PMC3972436

[ref9] Czyz, E. K., Horwitz, A. G., Arango, A., & King, C. A. (2019). Short-term change and prediction of suicidal ideation among adolescents: A daily diary study following psychiatric hospitalization. Journal of Child Psychology and Psychiatry and Allied Disciplines, 60(7), 732–741. 10.1111/jcpp.12974 30246870 PMC6726492

[ref10] Davidson, C. L., Anestis, M. D., & Gutierrez, P. M. (2017). Ecological momentary assessment is a neglected methodology in suicidology. Archives of Suicide Research, 21(1), 1–11. 10.1080/13811118.2015.1004482 26821811

[ref11] de Beurs, D. (2017). Network analysis: A novel approach to understand suicidal behaviour. International Journal of Environmental Research and Public Health, 14(3), 219. 10.3390/ijerph14030219

[ref12] de Beurs, D., Kirtley, O., Kerkhof, A., Portzky, G., & O'Connor, R. C. (2015). The role of mobile phone technology in understanding and preventing suicidal behavior. Crisis, 36(2), 79–82. 10.1027/0227-5910/a000316 30249123

[ref13] de Beurs, D., Bockting, C., Kerkhof, A., Scheepers, F., O'Connor, R., Penninx, B., & van de Leemput, I. (2021). A network perspective on suicidal behavior: Understanding suicidality as a complex system. Suicide and Life-Threatening Behavior, 51(1), 115–126. 10.1111/sltb.12676 33624872 PMC7986393

[ref14] Díaz-Oliván, I., Porras-Segovia, A., Barrigón, M. L., Jiménez-Muñoz, L., & Baca-García, E. (2021). Theoretical models of suicidal behaviour: A systematic review and narrative synthesis. The European Journal of Psychiatry, 35(3), 181–192. 10.1016/j.ejpsy.2021.02.002

[ref15] Duncan, S., & Barrett, L. F. (2007). Affect is a form of cognition: A neurobiological analysis. Cognition & Emotion, 21(6), 1184–1211. 10.1080/02699930701437931 18509504 PMC2396787

[ref16] Epskamp, S., Cramer, A. O. J., Waldorp, L. J., Schmittmann, V. D., & Borsboom, D. (2012). Qgraph: Network visualizations of relationships in psychometric data. Journal of Statistical Software, 48(4), 1–18. 10.18637/jss.v048.i04

[ref17] Epskamp, S., Deserno, M. K., & Bringmann, L. F. (2021). mlVAR: Multi-level vector autoregression [R package version 0.5.1]. Retrieved from: https://cran.r-project.org/web/packages/mlVAR/mlVAR.pdf

[ref18] Franklin, J. C., Ribeiro, J. D., Fox, K. R., Bentley, K. H., Kleiman, E. M., Huang, X., … Nock, M. K. (2017). Risk factors for suicidal thoughts and behaviors: A meta-analysis of 50 years of research. Psychological Bulletin, 143(2), 187–232. 10.1037/bul0000084 27841450

[ref19] Fried, E. I. (2022). Studying mental health problems as systems, not syndromes. Current Directions in Psychological Science, 31(6), 500–508. 10.1177/09637214221114089

[ref20] Fried, E. I., & Cramer, A. O. J. (2017). Moving forward: Challenges and directions for psychopathological network theory and methodology. Perspectives on Psychological Science, 12(6), 999–1020. 10.1177/1745691617705892 28873325

[ref21] Fried, E. I., & Robinaugh, D. J. (2020). Systems all the way down: Embracing complexity in mental health research. BMC Medicine, 18(1), 205. 10.1186/s12916-020-01668-w 32660482 PMC7359484

[ref22] Glenn, C. R., & Nock, M. K. (2014). Improving the short-term prediction of suicidal behavior. American Journal of Preventive Medicine, 47(3 Suppl. 2), S176–S180. 10.1016/j.amepre.2014.06.004 25145736 PMC5258198

[ref23] Goffnett, J., Liechty, J. M., & Kidder, E. (2020). Interventions to reduce shame: A systematic review. Journal of Behavioral and Cognitive Therapy, 30(2), 141–160. 10.1016/j.jbct.2020.03.001

[ref24] Goldston, D. B., Erkanli, A., Daniel, S. S., Heilbron, N., Weller, B. E., & Doyle, O. (2016). Developmental trajectories of suicidal thoughts and behaviors from adolescence through adulthood. Journal of the American Academy of Child & Adolescent Psychiatry, 55(5), 400–407.e1. 10.1016/j.jaac.2016.02.010 27126854 PMC5035543

[ref25] Hallard, R. I., Wells, A., Aadahl, V., Emsley, R., & Pratt, D. (2021). Metacognition, rumination and suicidal ideation: An experience sampling test of the self-regulatory executive function model. Psychiatry Research, 303, 114083. 10.1016/j.psychres.2021.114083 34271370

[ref26] Hallensleben, N., Glaesmer, H., Forkmann, T., Rath, D., Strauss, M., Kersting, A., & Spangenberg, L. (2019). Predicting suicidal ideation by interpersonal variables, hopelessness and depression in real-time: An ecological momentary assessment study in psychiatric inpatients with depression. European Psychiatry, 56, 43–50. 10.1016/j.eurpsy.2018.11.003 30530103

[ref27] Howarth, E. J., O'Connor, D. B., Panagioti, M., Hodkinson, A., Wilding, S., & Johnson, J. (2020). Are stressful life events prospectively associated with increased suicidal ideation and behaviour? A systematic review and meta-analysis. Journal of Affective Disorders, 266, 731–742. 10.1016/j.jad.2020.01.171 32217256

[ref28] Husky, M., Swendsen, J., Ionita, A., Jaussent, I., Genty, C., & Courtet, P. (2017). Predictors of daily life suicidal ideation in adults recently discharged after a serious suicide attempt: A pilot study. Psychiatry Research, 256, 79–84. 10.1016/j.psychres.2017.06.035 28624676

[ref29] Jongeneel, A., Aalbers, G., Bell, I., Fried, E. I., Delespaul, P., Riper, H., … van den Berg, D. (2020). A time-series network approach to auditory verbal hallucinations: Examining dynamic interactions using experience sampling methodology. Schizophrenia Research, 215, 148–156. 10.1016/j.schres.2019.10.055 31780345

[ref30] Kaurin, A., Dombrovski, A. Y., Hallquist, M. N., & Wright, A. G. C. (2020). Momentary interpersonal processes of suicidal surges in borderline personality disorder. Psychological Medicine, 52(13), 2702–2712. 10.1017/S0033291720004791 33298227 PMC8190164

[ref31] Kivelä, L., van der Does, W. A. J., Riese, H., & Antypa, N. (2022). Don't miss the moment: A systematic review of ecological momentary assessment in suicide research. Frontiers in Digital Health, 4, 876595. 10.3389/fdgth.2022.876595 35601888 PMC9120419

[ref32] Kivelä, L. M. M., Fiß, F., van der Does, W., & Antypa, N. (2023). Examination of acceptability, feasibility and iatrogenic effects of ecological momentary assessment (EMA) of suicidal ideation. Assessment, 10731911231216053. 10.1177/10731911231216053 PMC11292966 38098238

[ref33] Kleiman, E. M., Law, K. C., & Anestis, M. D. (2014). Do theories of suicide play well together? Integrating components of the hopelessness and interpersonal psychological theories of suicide. Comprehensive Psychiatry, 55(3), 431–438. 10.1016/j.comppsych.2013.10.015 24332385

[ref34] Kleiman, E. M., Turner, B. J., Fedor, S., Baele, E. E., Huffman, J. C., & Nock, M. K. (2017). Examination of real-time fluctuations in suicidal ideation and its risk factors: Results from two ecological momentary assessment studies. Journal of Abnormal Psychology, 126(6), 726–738. 10.1037/abn0000273.supp 28481571

[ref35] Kleiman, E. M., Coppersmith, D. D. L., Millner, A. J., Franz, P. J., Fox, K. R., & Nock, M. K. (2018). Are suicidal thoughts reinforcing? A preliminary real-time monitoring study on the potential affect regulation function of suicidal thinking. Journal of Affective Disorders, 232, 122–126. 10.1016/j.jad.2018.02.033 29481996

[ref36] Kwiatkowski, D., Phillips, P. C. B., Schmidt, P., & Shin, Y. (1992). Testing the null hypothesis of stationarity against the alternative of a unit root. Journal of Econometrics, 54(1–3), 159–178. 10.1016/0304-4076(92)90104-Y

[ref37] Lester, D. (1997). The role of shame in suicide. Suicide and Life-Threatening Behavior, 27(4), 352–361. 9444730

[ref38] Littlewood, D. L., Kyle, S. D., Carter, L. A., Peters, S., Pratt, D., & Gooding, P. (2019). Short sleep duration and poor sleep quality predict next-day suicidal ideation: An ecological momentary assessment study. Psychological Medicine, 49(3), 403–411. 10.1017/S0033291718001009 29697037 PMC6331731

[ref39] Nock, M. K. (2016). Recent and needed advances in the understanding, prediction, and prevention of suicidal behavior. Depression and Anxiety, 33(6), 460–463). 10.1002/da.22528 27248363

[ref40] Nock, M. K., Borges, G., Bromet, E. J., Alonso, J., Angermeyer, M., Beautrais, A., … Williams, D. (2008). Cross-national prevalence and risk factors for suicidal ideation, plans and attempts. British Journal of Psychiatry, 192(2), 98–105. 10.1192/bjp.bp.107.040113 PMC2259024 18245022

[ref41] Nock, M. K., Pristein, M. J., & Sterba, S. K. (2009). Revealing the form and function of self-injurious thoughts and behaviors: A real-time ecological assessment study among adolescents and young adults. Journal of Abnormal Psychology, 118(4), 816–827. 19899851 10.1037/a0016948 PMC5258190

[ref42] Nyström, M. B. T., Kjellberg, E., Heimdahl, U., & Jonsson, B. (2018). Shame and interpersonal sensitivity: Gender differences and the association between internalized shame coping strategies and interpersonal sensitivity. Bulletin of the Menninger Clinic, 82(2), 137–155. 10.1521/bumc.2018.82.2.137 29791193

[ref43] Posner, K., Brown, G. K., Stanley, B., Brent, D. A., Yershova, K. V., Oquendo, M. A., … Mann, J. J. (2011). The Columbia–Suicide Severity Rating Scale: Initial validity and internal consistency findings from three multisite studies with adolescents and adults. American Journal of Psychiatry, 168(12), 1266–1277. 10.1176/appi.ajp.2011.10111704 22193671 PMC3893686

[ref44] Rath, D., de Beurs, D., Hallensleben, N., Spangenberg, L., Glaesmer, H., & Forkmann, T. (2019). Modelling suicide ideation from beep to beep: Application of network analysis to ecological momentary assessment data. Internet Interventions, 18, 100292. 10.1016/j.invent.2019.100292 31828015 PMC6889482

[ref45] Rudd, M. D. (2008). Suicide warning signs in clinical practice. Current Psychiatry Reports, 10(1), 87–90. 10.1007/s11920-008-0015-4 18269900

[ref46] Rudd, M. D., Berman, A. L., Joiner, T. E., Nock, M. K., Silverman, M. M., Mandrusiak, M., … Witte, T. (2006). Warning signs for suicide: Theory, research, and clinical applications. Suicide and Life-Threatening Behavior, 36(3), 255–262. 10.1521/suli.2006.36.3.255 16805653

[ref47] Sani, G., Tondo, L., Koukopoulos, A., Reginaldi, D., Kotzalidis, G. D., Koukopoulos, A. E., … Tatarelli, R. (2011). Suicide in a large population of former psychiatric inpatients. Psychiatry and Clinical Neurosciences, 65(3), 286–295. 10.1111/j.1440-1819.2011.02205.x 21507136

[ref48] Sheehan, D. V, Lecrubier, Y., Sheehan, K. H., Amorim, P., Janavs, J., Weiller, E., … Dunbar, G. C. (1998). The Mini-International Neuropsychiatric Interview (M.I.N.I.): The development and validation of a structured diagnostic psychiatric interview for DSM-IV and ICD-10. The Journal of Clinical Psychiatry, 59 Suppl. 20, 22-33. 9881538

[ref49] Sheehy, K., Noureen, A., Khaliq, A., Dhingra, K., Husain, N., Pontin, E. E., … Taylor, P. J. (2019). An examination of the relationship between shame, guilt and self-harm: A systematic review and meta-analysis. Clinical Psychology Review, 73, 101779. 10.1016/j.cpr.2019.101779 31707184 PMC6891258

[ref50] Shiffman, S., Stone, A. A., & Hufford, M. R. (2008). Ecological momentary assessment. Annual Review of Clinical Psychology, 4(1), 1–32. 10.1146/annurev.clinpsy.3.022806.091415 18509902

[ref51] Spangenberg, L., Glaesmer, H., Hallensleben, N., Rath, D., & Forkmann, T. (2019). (In)stability of capability for suicide in psychiatric inpatients: Longitudinal assessment using ecological momentary assessments. Suicide and Life-Threatening Behavior, 49(6), 1560–1572. 10.1111/sltb.12547 30834576

[ref52] Stanley, B., Martínez-Alés, G., Gratch, I., Rizk, M., Galfalvy, H., Choo, T. H., & Mann, J. J. (2021). Coping strategies that reduce suicidal ideation: An ecological momentary assessment study. Journal of Psychiatric Research, 133, 32–37. 10.1016/j.jpsychires.2020.12.012 33307352 PMC8659118

[ref53] Van Orden, K. A., Witte, T. K., Cukrowicz, K. C., Braithwaite, S. R., Selby, E. A., & Joiner, T. E. (2010). The interpersonal theory of suicide. Psychological Review, 117(2), 575–600. 10.1037/a0018697 20438238 PMC3130348

[ref54] Van Orden, K. A., Talbot, N., & King, D. (2012). Using the Interpersonal Theory of Suicide to inform interpersonal psychotherapy with a suicidal older adult. Clinical Case Studies, 11(5), 333–347. 10.1177/1534650112457710 27087791 PMC4830505

[ref55] Veenman, M., Janssen, L. H. C., Van Houtum, L. A. E. M., Wever, M. C. M., Verkuil, B., Epskamp, S., … Elzinga, B. M. (2024). A network study of family affect systems in daily life. Multivariate Behavioral Research, 59(2), 371–405. 10.1080/00273171.2023.2283632 38356299

[ref56] Victor, S. E., Scott, L. N., Stepp, S. D., & Goldstein, T. R. (2019). I want you to want me: Interpersonal stress and affective experiences as within-person predictors of non-suicidal self-injury and suicide urges in daily life. Suicide and Life-Threatening Behavior, 49(4), 1157–1177. 10.1111/sltb.12513 30159910 PMC6395579

[ref57] Wastler, H. M., Khazem, L. R., Ammendola, E., Baker, J. C., Bauder, C. R., Tabares, J., … Bryan, C. J. (2023). An empirical investigation of the distinction between passive and active ideation: Understanding the latent structure of suicidal thought content. Suicide and Life-Threatening Behavior, 53(2), 219–226. 10.1111/sltb.12935 36504400

