# Supplementary material for: Examining contemporaneous and temporal associations of real-time suicidal ideation using network analysis
Source: Psychol Med. 2024 Sep 9;54(12):3357–65. doi: 10.1017/S003329172400151X (PMC11496231; doi:10.1017/S003329172400151X)
Supplement: Kivelä et al. supplementary material [file S003329172400151Xsup001.docx]

R version 4.0.2 (2020-06-22)

Platform: x86_64-apple-darwin17.0 (64-bit)

Running under: OS X 11.4

Matrix products: default

LAPACK: /Library/Frameworks/R.framework/Versions/4.0/Resources/lib/libRlapack.dylib

locale:

[1] en_US.UTF-8/en_US.UTF-8/en_US.UTF-8/C/en_US.UTF-8/en_US.UTF-8

attached base packages:

[1] stats graphics grDevices

[4] utils datasets methods

[7] base

other attached packages:

[1] psych_1.9.12.31 qgraph_1.6.5

[3] dplyr_1.0.7 foreign_0.8-81

[5] mlVAR_0.5.1

loaded via a namespace (and not attached):

[1] nlme_3.1-151

[2] xts_0.12.1

[3] httr_1.4.5

[4] RColorBrewer_1.1-2

[5] tools_4.0.2

[6] backports_1.1.8

[7] utf8_1.1.4

[8] R6_2.4.1

[9] rpart_4.1-15

[10] d3Network_0.5.2.1

[11] Hmisc_4.4-2

[12] colorspace_1.4-1

[13] nnet_7.3-14

[14] tidyselect_1.1.0

[15] gridExtra_2.3

[16] mnormt_2.0.0

[17] curl_4.3

[18] compiler_4.0.2

[19] fdrtool_1.2.15

[20] cli_2.0.2

[21] glmnet_4.0-2

[22] htmlTable_2.0.1

[23] tseries_0.10-47

[24] scales_1.1.1

[25] checkmate_2.0.0

[26] mvtnorm_1.1-1

[27] quadprog_1.5-8

[28] pbapply_1.4-2

[29] stringr_1.4.0

[30] digest_0.6.25

[31] pbivnorm_0.6.0

[32] minqa_1.2.4

[33] base64enc_0.1-3

[34] jpeg_0.1-8.1

[35] pkgconfig_2.0.3

[36] htmltools_0.5.0

[37] lme4_1.1-26

[38] htmlwidgets_1.5.1

[39] rlang_0.4.11

[40] TTR_0.24.2

[41] rstudioapi_0.14

[42] quantmod_0.4.17

[43] huge_1.3.4.1

[44] shape_1.4.5

[45] generics_0.0.2

[46] zoo_1.8-8

[47] gtools_3.8.2

[48] graphicalVAR_0.2.4

[49] magrittr_2.0.3

[50] Formula_1.2-3

[51] texreg_1.37.5

[52] Matrix_1.2-18

[53] Rcpp_1.0.4.6

[54] munsell_0.5.0

[55] fansi_0.4.1

[56] proto_1.0.0

[57] abind_1.4-5

[58] lifecycle_1.0.0

[59] stringi_1.4.6

[60] whisker_0.4

[61] clusterGeneration_1.3.6

[62] MASS_7.3-51.6

[63] plyr_1.8.6

[64] lavaan_0.6-6

[65] grid_4.0.2

[66] parallel_4.0.2

[67] crayon_1.3.4

[68] lattice_0.20-41

[69] splines_4.0.2

[70] pander_0.6.3

[71] tmvnsim_1.0-2

[72] knitr_1.29

[73] pillar_1.6.1

[74] igraph_1.2.5

[75] boot_1.3-25

[76] rjson_0.2.20

[77] corpcor_1.6.9

[78] BDgraph_2.62

[79] codetools_0.2-16

[80] reshape2_1.4.4

[81] stats4_4.0.2

[82] glue_1.6.2

[83] latticeExtra_0.6-29

[84] data.table_1.12.8

[85] foreach_1.5.1

[86] png_0.1-7

[87] vctrs_0.3.8

[88] nloptr_1.2.2.2

[89] gtable_0.3.0

[90] purrr_0.3.4

[91] assertthat_0.2.1

[92] gsubfn_0.7

[93] ggplot2_3.3.2

[94] xfun_0.15

[95] xtable_1.8-4

[96] coda_0.19-3

[97] survival_3.1-12

[98] glasso_1.11

[99] tibble_3.0.1

[100] arm_1.11-1

[101] iterators_1.0.13

[102] MplusAutomation_0.8

[103] cluster_2.1.0

[104] statmod_1.4.34

[105] ellipsis_0.3.2
